# Sodium nitroprusside restored lipopolysaccharide‐induced learning and memory impairment in male rats via attenuating inflammation and oxidative stress

**DOI:** 10.14814/phy2.16053

**Published:** 2024-05-28

**Authors:** Zeinab Hosseini, Farimah Beheshti, Faezeh Sadat Hosseini Kakhki, Mahmoud Hosseini, Akbar Anaeigoudari

**Affiliations:** ^1^ Applied Biomedical Research Center Mashhad University of Medical Sciences Mashhad Iran; ^2^ Neuroscience Research Center Torbat Heydariyeh University of Medical Sciences Torbat Heydariyeh Iran; ^3^ Department of Physiology, School of Paramedical Sciences Torbat Heydariyeh University of Medical Sciences Torbat Heydariyeh Iran; ^4^ Psychiatry and Behavioral Sciences Research Center Mashhad University of Medical Sciences Mashhad Iran; ^5^ Department of Physiology, School of Medicine Jiroft University of Medical Sciences Jiroft Iran

**Keywords:** inflammation, lipopolysaccharide, oxidative damage, sodium nitroprusside

## Abstract

Inflammation and oxidative stress upset memory. We explored influence of sodium nitroprusside (SNP) on memory deficits resulted from lipopolysaccharide (LPS).Groups include control, LPS, LPS + SNP 1 mg/kg, LPS + SNP 2 mg/kg, and LPS + SNP 3 mg/kg. Morris water maze and passive avoidance tests and biochemical measurements were carried out.In Morris water maze, LPS prolonged time and distance for finding the platform. In probe trial, it diminished time spent and traveled distance in the target zone. Injection of 2 and 3 mg/kg of SNP overturned the effect of LPS. In passive avoidance task, LPS postponed entrance into darkroom and reduced time spent in light room and incremented time spent in darkroom in 3, 24, and 72 h after electrical shock. All three doses of SNP restored the effects of LPS. Biochemical experiments confirmed that LPS elevated interleukin‐6 and malondialdehyde concentration and declined total thiol content and superoxide dismutase and catalase activity in the hippocampus and cortex tissues. SNP particularly at a 3 mg/kg dose ameliorated LPS effects on these parameters.SNP attenuated memory disabilities resulting from LPS through modifying inflammation and boosting antioxidant defense.

## Graphical abstract

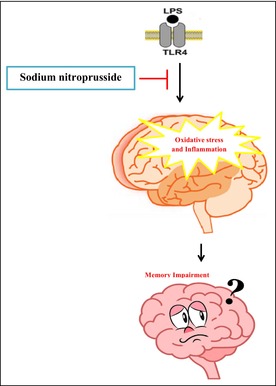

## 1 INTRODUCTION

Inflammation is a cellular response resulting from exogenous and endogenous causes (Sousa et al., 2020). Microbial infections, allergens, poisons, and tissue damages can elicit inflammatory reactions (Grahnert et al., 2019). Along with the positive role of inflammation in establishing homeostasis, chronic inflammation has a basic contributor in inducing of brain disorders and cognitive deficits (Ghasemi‐Tarie et al., 2022). Researchers reported that uncontrolled release of inflammatory factors including interleukin (IL)‐1β disturb long‐term memory (Barrientos et al., 2002). It has been also cleared that acute systemic inflammation attenuates hippocampal‐dependent working memory (Murray et al., 2012). Meantime, a pile of evidence supports the destructive role of oxidative stress on learning and memory (Fukui et al., 2001). Furthermore, there is a two‐way relationship between inflammatory reactions and oxidative injuries (Zhu et al., 2022). It has been recognized that inflammatory mediators enhance the generation of oxidative agents by immune cells. On the other hand, injuries resulted from over generation of reactive oxygen species (ROS) has been reported to evoke the nuclear factor κB (NFκB) pathway and inflammation (Federico et al., 2007).

Lipopolysaccharide (LPS), a major component of the cell wall of bacteria, stimulates the generation of inflammatory cytokines which in turn induces oxidative stress (Abareshi et al., 2016; Hosseini et al., 2017). Infusion of LPS into the brain ventricles has been suggested to trigger the inflammation of the hippocampus and entorhinal cortex tissue (Hauss‐Wegrzyniak et al., 2002). Researchers also declared that systemic inflammation and oxidative damage resulting from LPS induce anxiety and depression like behaviors and impair spatial learning and memory retention in rodents (Anaeigoudari et al. 2016; Azizi‐Malekabadi et al., 2015). Reports exhibited that administration of LPS into the brain increased tumor necrosis factor (TNF)‐α expression and inhibited hippocampal‐dependent long‐term potentiation (LTP) induction (Abareshi et al., 2016; Tancredi et al., 1992).

Sodium nitroprusside (SNP) with chemical structure Na_2_ [Fe(CN)5NO] can release nitric oxide (NO) or NO‐related species. Therefore, it is named a NO donor (Ortuño‐Sahagún, 2013). NO donors have anti‐inflammatory properties, and down‐regulate adhesion molecules in the central nervous system (Prasad et al., 2007). The protective effects of SNP against damages induced by inflammation in various organs have been also demonstrated. For example, SNP attenuated the generation of inflammatory mediators in the kidney and lung in inflammation followed by ischemia–reperfusion (Ortuño‐Sahagún, 2013). It has been also documented that SNP suppressed LPS‐caused cyclooxygenase 2 (COX‐2) expression and reduced oxidative damage in the brain ischemia (D'Acquisto et al., 2001). This study investigates the effect of SNP on LPS‐induced learning and memory deficits.

## 2 MATERIALS AND METHODS

**FIGURE 1:**
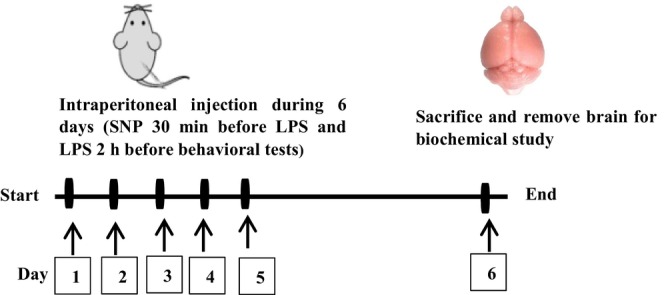
A schematic timeline of type and sequence of experiments.

Forty adult male Wistar rats were taken from laboratory breeding stock in Mashhad University of Medical Sciences, Mashhad, Iran. The animals were kept in a regulated temperature (22 ± 2°C), a light/dark cycle 12/12 and adequate humidity. The rats also freely received food (Javaneh Khorasan Company) and water. The experimental groups included as follows: (1) control (2) LPS, (3) LPS + SNP 1 mg/kg, (4) LPS + SNP 2 mg/kg, and (5) LPS + SNP 3 mg/kg (*n* = 7–8 in each group). Control group received saline. Other groups were treated with LPS (1 mg/kg). The LPS + SNP groups also received 1, 2, and 3 mg/kg of SNP. LPS injection happened 2 h prior to the behavioral tests. The LPS + SNP groups received SNP 30 min before LPS. All treatments were done intraperitoneally. Figure 1 demonstrates a schematic timeline of type and sequence of experiments. The experiments were conducted based on the structures submitted by Ethical Committee of Mashhad University of Medical Sciences (IR.MUMS.MEDICAL.REC.1400.094). Preparation of LPS (*E. coli* 055:B5, L2880) and SNP (Sigma Aldrich Company, United States, Catalog No. 13755–38‐9) were done from Sigma. Reagents used for biochemical tests were provided from Merck Company. The level of IL‐6 was checked by a specific ELISA kit.

### 2.1 Morris water maze test

The Morris water maze (MWM) is an apparatus for studying spatial learning and memory. For this purpose, the rats are released in a circular pool of water and admitted to find a platform hidden under surface of the water. Training sessions were performed for 5 days. On the sixth day, the platform was thrown out and probe test was done. A video camera was hanged above of the center of the pool to record the images of swimming animals. Time of swimming and traveled path by rats were compared between groups (Hosseini et al., 2010; Saffarzadeh et al., 2010).

### 2.2 Passive avoidance test

Passive avoidance (PA) is a device by which the animal is trained to avoid a natural response. In PA, the animal entered from a lightened room to a darkened chamber. Upon entry to the darkened room, the animal was punished by an electrical shock. Recall test was performed 3, 24, and 72 h after the training session. Measured parameters included delay to enter the darkened room and time spent in darkened and lightened rooms (Beheshti et al., 2017).

### 2.3 Biochemical assessments

At the end of behavioral experiments, the rats were profoundly anesthetized by urethane (1.2 g/kg). Eventually, the animals were sacrificed and hippocampi and cortex tissues were gathered. Then, the supernatants of homogenized specimens were isolated and used for checking the biochemical parameters.

#### 2.3.1 Determination of malondialdehyde (MDA) and total thiol groups

Thiobarbituric acid (TBA, Sigma Aldrich Company, United States, Catalog No: T5500) was used for the determination of MDA level of hippocampi and cortex tissues. The absorbance of red colored complex was read at 535 nm. 2 mL of TBA was mixed with 1 mL of homogenized samples and heated. Then, the absorbance of cooled solution was measured at 535 nm. 5,5′‐dithiobis(2‐nitrobenzoic acid) (DTNB, Sigma Aldrich Company, United States, Catalog No: 103291) as a reagent was used to determine the total thiol groups content of brain tissues. The absorbance of colored complex resulting from the reaction of DTNB with the total thiol groups was monitored at 412 nm.

#### 2.3.2 The enzymatic assays

The evaluation of superoxide dismutase (SOD) activity was accomplished by a colorimetric method in which tetrazolium dye MTT (3‐(4, 5‐dimethylthiazol‐2‐yl) 2, 5‐diphenyltetrazolium bromide, Sigma Aldrich Company, United States, Catalog No: 475989) is reduced to colored formazan. The amount of reduction of MTT illustrates the level of superoxide production (Madesh & Balasubramanian, 1998). Catalase (CAT) activity was indicated by ultraviolet spectrophotometric method. In this protocol, dissociation of hydrogen peroxidase into oxygen and water was perceived as an alteration in absorbance at 240 nm (Aebi, 1984).

#### 2.3.3 Determination of hippocampal IL‐6 content

An ELISA kit (eBioscince BMS625 Rat IL‐6 Platinum ELISA) was employed to assess the IL‐6 level. Measurement was achieved based on instruction of company.

#### 2.3.4 Statistical analysis

Data of PA test were presented as median and interquartile range. In all other cases, means ± SD was used to express the data. For analyzing the gathered data during 5 days of MWM, repeated measures analysis of variance followed by the Bonferroni multiple comparison test was employed. The extracted data in probe day in MWM and biochemical tests were analyzed using one‐way ANOVA followed by Tukey's post hoc test. We analyzed the data of the PA test by Kruskal–Wallis followed by Dunn's pairwise comparison test. *p* < 0.05 was regarded as significant level.

## 3 RESULTS

### 3.1 Behavioral results

#### 3.1.1 MWM results

**FIGURE 2:**
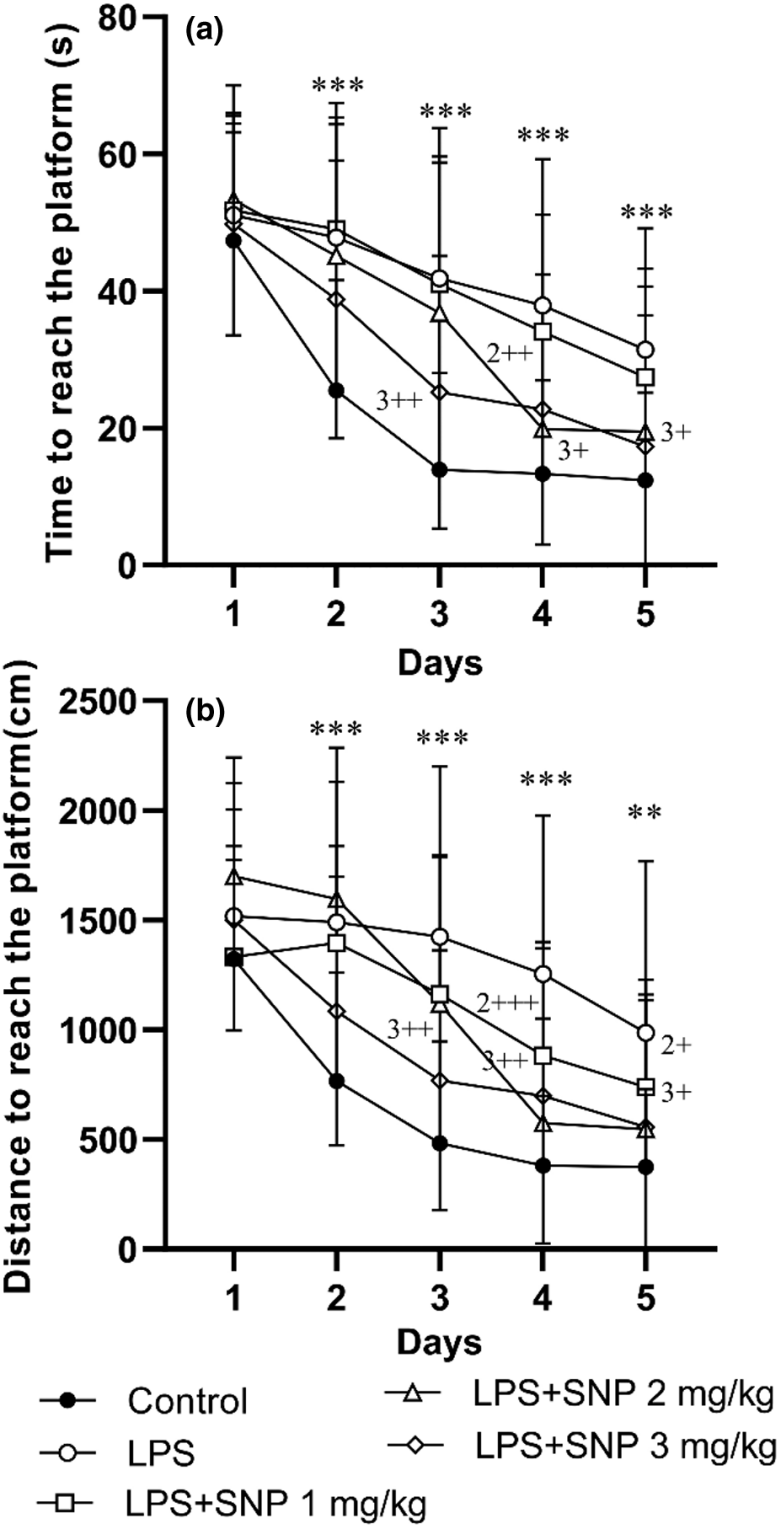
Comparison of time latency (a) and traveled distance (b) to find the platform in the Morris water maze between experimental groups. Data are presented as mean ± SD (*n* = 7–8 in each group). **p < 0.001 and ***p < 0.001 compared to control group. ^+^ *p* < 0.05, ^++^ *p* < 0.01 and ^+++^ *p* < 0.001 compared to LPS group, 2 = SNP 2 mg/kg, 3 = SNP 3 mg/kg.

The results exhibits a noticeable difference among the groups in time (*F*_(4,151)_ = 15.48, *p* < 0.001) and distance (*F*_(4,151)_ = 14.29, *p* < 0.001) to reach the platform. Data also specified a main effect for day on time (*F*_(4,604)_ = 87.14, *p* < 0.001) and distance (*F*_(4,604)_ = 71.99, *p* < 0.001) to reach the platform. We also saw an interaction for treatment × day (*F*_(16,604)_ = 2.73, *p* < 0.001 for time and *F*_(16,604)_ = 3.82, *p* < 0.001 for distance). Further analysis confirmed that the rats treated by LPS spent more time and traveled more distance for reaching the hidden platform compared to control group when they were tested by MWM test (*p* < 0.01–*p* < 0.001). Injection of 2 and 3 mg/kg of SNP resulted to a significant decrease in time spent and traveled distance to find the platform by rats of LPS‐SNP 2 mg/kg and LPS‐SNP 3 mg/kg groups versus LPS group (*p* < 0.05–*p* < 0.001) (Figure 2a,b).

**FIGURE 3:**
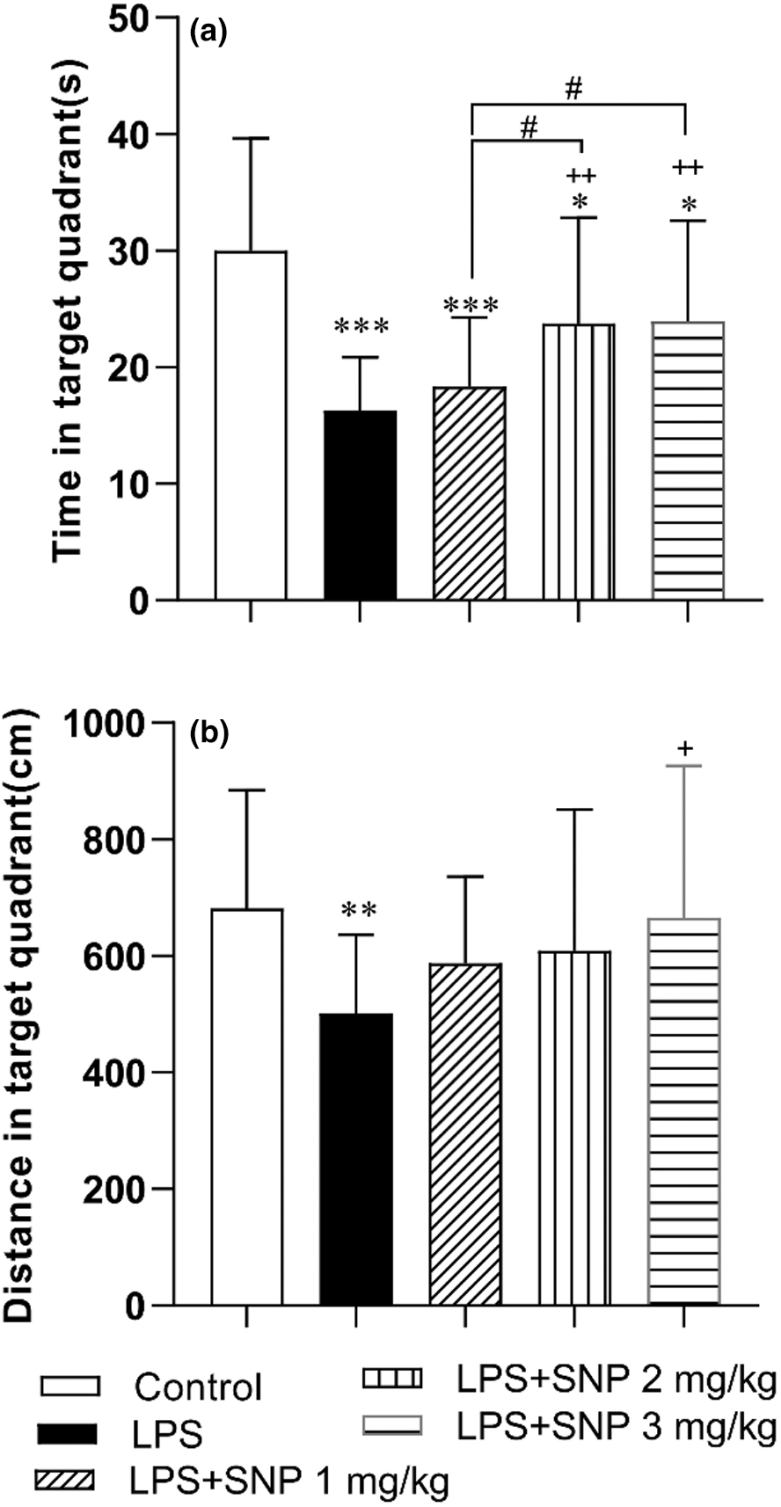
The result of the time (a) and distance (b) in the target quadrant Morris water maze between experimental groups in target quadrant in probe day. The platform was removed and the time spent and distance traveled in the target quadrant was recorded and compared between groups. Data are shown as mean ± SD (*n* = 7–8 in each group). *p < 0.05, **p < 0.01 and ***p < 0.001 compared to control group and ^+^ *p* < 0.05 and ^++^ *p* < 0.01 compared to LPS group #*p* < 0.05 compared to LPS+SNP 1 mg/kg.

The results of the probe day indicated that a significant difference among the groups in traveling time and distance in the target area of MWM (*F*_(4,151)_ = 14.40, *p* < 0.001 for time and *F*_(4,151)_ = 3.80, *p* < 0.01 for distance). Further analysis showed that the time spent and traveled distance in target quadrant in LPS group was shorter than control group (*p* < 0.01 and *p* < 0.001). Treatment with SNP prolonged the searching time in target quadrant in LPS‐SNP 2 mg/kg and LPS‐SNP 3 mg/kg groups versus LPS group (both *p* < 0.01). LPS‐SNP 3 mg/kg group also traveled more distance in target quadrant than LPS group (*p* < 0.01). The rats of LPS‐SNP 2 mg/kg and PS‐SNP 3 mg/kg groups spent more time in target quadrant than LPS‐SNP 1 mg/kg group (*p* < 0.05) (Figure 3a,b).

#### 3.1.2 Passive avoidance results

**FIGURE 4:**
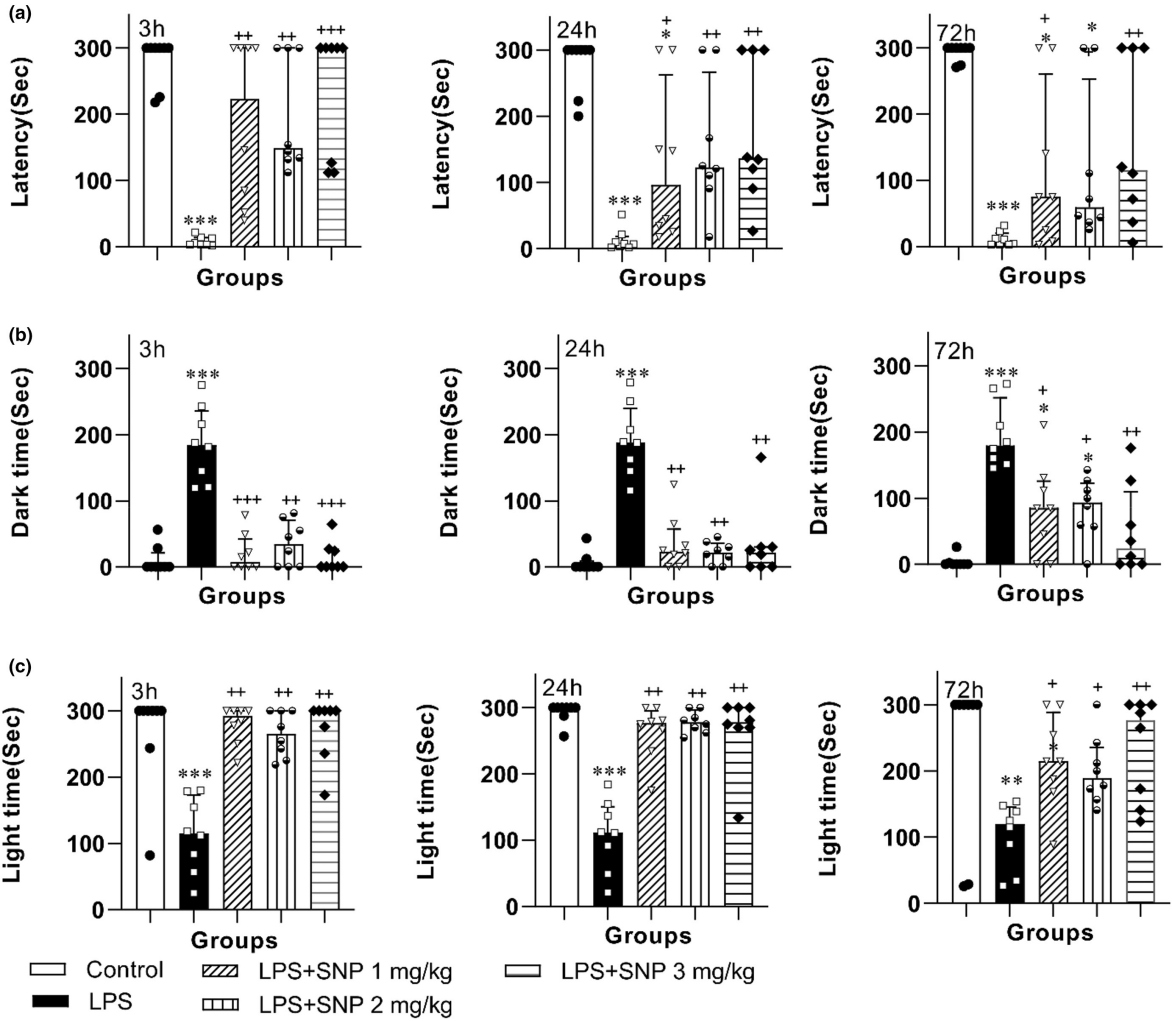
Comparison of time latency to enter the dark room (a) and the total time spent in dark room (b) and the total time spent in light room (c) at the 3, 24, and 72 h after receiving shock in the passive avoidance test in the experimental groups. The data are presented as median and interquartile range (*n* = 7–8). *p < 0.01, **p < 0.01 and ***p < 0.001 compared to control group. ^+^ *p* < 0.05, ^++^ *p* < 0.01 and ^+++^ *p* < 0.001 compared to LPS group.

Data analysis by Kruskal–Wallis test highlighted difference in the latency time among the groups at 3 h (*H*_(4)_ = 22.68; *p* < 0.001), 24 h (*H*_(4)_ = 22.56; *p* < 0.001), and 48 h (*H*_(4)_ = 20.61; *p* < 0.001) after applying the shock. The results of PA also indicated that the delay to enter the darkened room at 3, 24, and 72 h after exerting shock was shortened in LPS group versus control group (all *p* < 0.001). The time latency to enter the darkened chamber at 3, 24, and 72 h after applying shock in LPS‐SNP 1 mg/kg, LPS‐SNP 2 mg/kg and LPS‐SNP 3 mg/kg groups was longer than LPS group (*p* < 0.05–*p* < 0.001) (Figure 4a).

The results of Kruskal–Wallis test support the difference in the total time spent in the darkened room among the groups at 3 h (*H*_(4)_ = 22.29; *p* < 0.001), 24 h (*H*_(4)_ = 21.40; *p* < 0.001), and 72 h (*H*_(4)_ = 23.02; *p* < 0.001) after applying the shock. Subsequent of analysis showed that the total time spent in darkened compartment at 3, 24, and 72 h after delivering shock in the animals challenged by LPS significantly was longer than control group (all *p* < 0.001). The total time spent in darkened room at 3, 24, and 72 h after delivering shock in LPS‐SNP 1 mg/kg, LPS‐SNP 2 mg/kg and LPS‐SNP 3 mg/kg groups was lower than LPS group (*p* < 0.05–*p* < 0.001) (Figure 4b).

Kruskal–Wallis test approved difference in the total time spent in the light compartment among the groups at 3 h (*H*_(4)_ = 18.88; *p* < 0.001), 24 h (*H*_(4)_ = 21.40; *p* < 0.001), and 72 h (*H*_(4)_ = 12.55; *p* < 0.01) after applying the shock. Further analysis showed that LPS could reduce time spent in light chamber at 3, 24 and 72 h after exerting electrical shock in LPS group versus control group (*p* < 0.01 and *p* < 0.001). Administration of SNP prolonged the time spent in lightened chamber at 3, 24, and 72 h after exerting electrical shock in LPS–SNP groups with respect to LPS group (*p* < 0.05–*p* < 0.01) (Figure 4c).

### 3.2 Biochemical results

#### 3.2.1 Results of IL‐6 in the hippocampi

**FIGURE 5:**
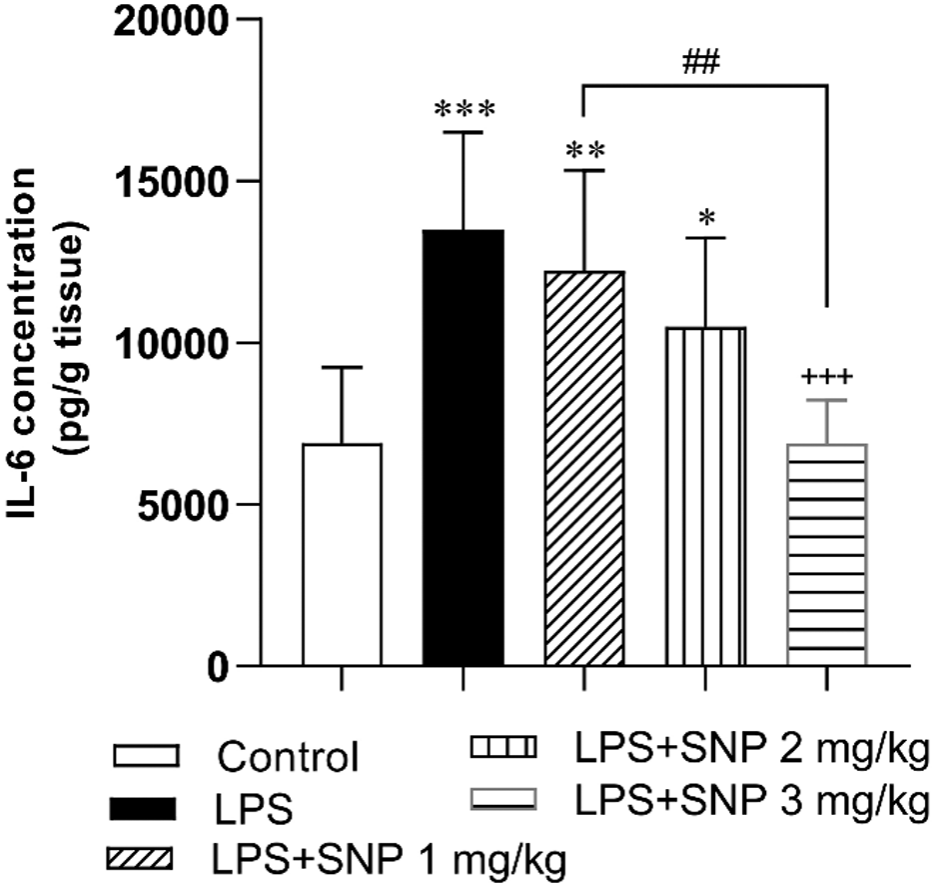
Comparison of the hippocampi IL‐6 content between experimental groups. Data are shown as mean ± SD (*n* = 7–8 in each group). *p < 0.05, **p < 0.01 and ***p < 0.001 compared to control group. ^+++^ *p* < 0.001 compared to LPS group. ^##^ *p* < 0.01 compared to the other treatment groups.

The findings of IL‐6 revealed a significant difference among the groups (*F*_(4,34)_ = 10.49, *p* < 0.001). Further analysis displayed that LPS injection was followed by a decrease of IL‐6 in the hippocampi (*p* < 0.001). Treatment with 3 mg/kg of SNP attenuated IL‐6 (*p* < 0.001) but 1 and 2 mg/kg doses were not effective. In addition, IL‐6 level in the rats treated by 3 mg/kg reached to the control level but in the groups treated by 1 and 2 mg/kg dose of SNP, IL‐6 level was higher than the control (*p* < 0.01 and *p* < 0.05, respectively) (Figure 5).

#### 3.2.2 Results of MDA and thiol concentration and SOD and CAT activity in the hippocampi

**FIGURE 6:**
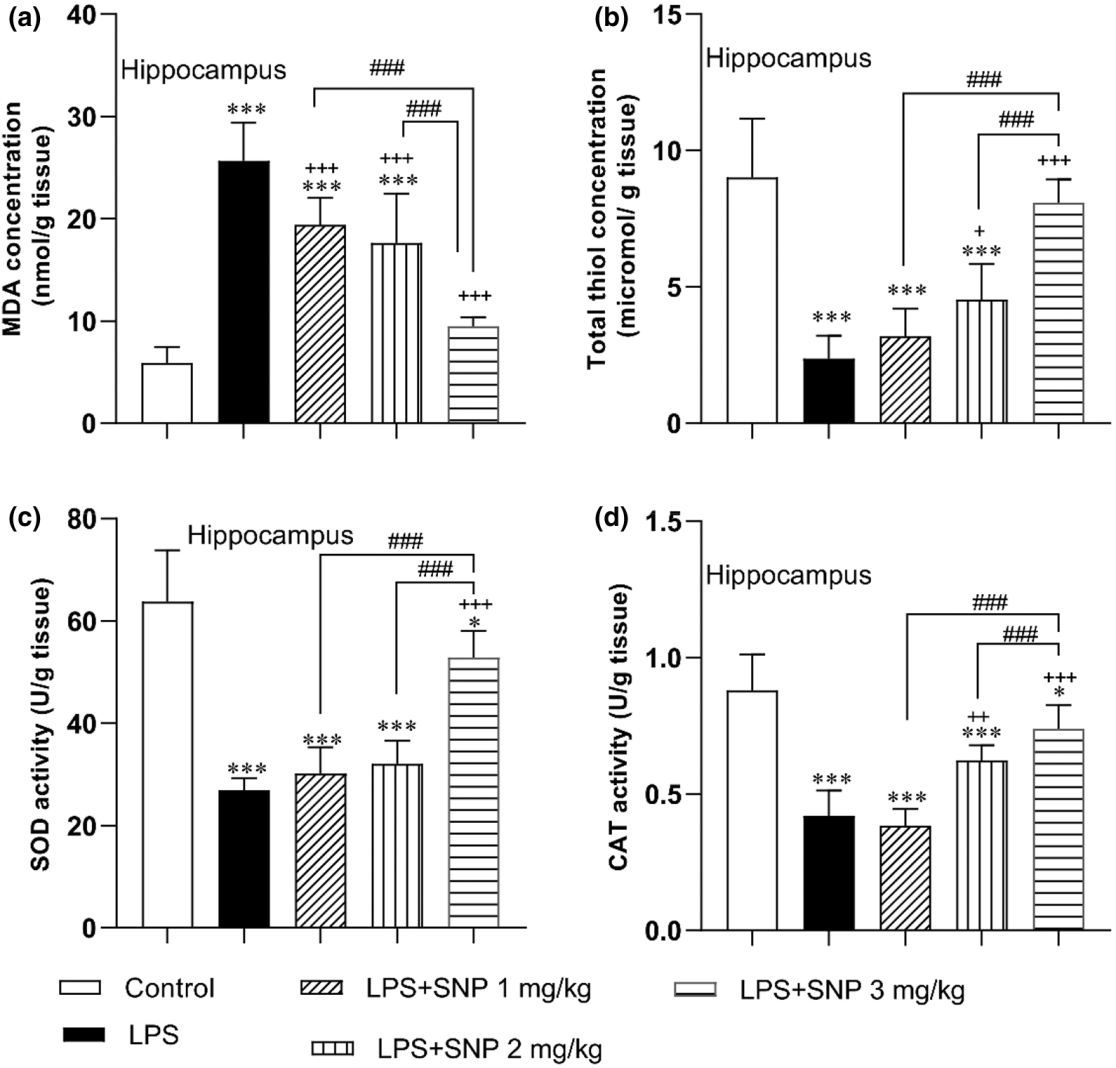
Comparison of the MDA (a) and thiol (b) concentration and SOD (c) and CAT (d) activity in hippocampi tissues between experimental groups. Data are presented as mean ± SD (*n* = 7 in each group). *p < 0.05 and ***p < 0.001 compared to control group. ^+^ *p* < 0.05, ^++^ *p* < 0.01, and ^+++^ *p* < 0.001 compared to LPS group. ^###^ *p* < 0.001 compared to the other treatment groups.

MDA concentration in hippocampi had a considerable difference among groups (*F*_(4,30)_ = 46.55, *p* < 0.001). The results also exhibited that LPS heightened the content of this product in hippocampi tissue of rats versus control group (*p* < 0.001). With respect to LPS group, the level of MDA in hippocampi tissue in LPS + SNP groups significantly declined (*p* < 0.001 for all three doses). Comparison of MDA level between the rats treated by SNP indicated that the content of this index in hippocampi tissue of LPS + SNP 3 mg/kg group was less than LPS + SNP 1 mg/kg and LPS–SNP 2 mg/kg groups (*p* < 0.001) (Figure 6a). The thiol level in the hippocampi had a considerable difference among groups (*F*_(4,30)_ = 34.83, *p* < 0.001). As illustrated in Figure 6b, LPS administration reduced the level of total thiol in the hippocampi of LPS group versus control group (*p* < 0.001). This antioxidant parameter significantly was increased in hippocampi tissue of rats in LPS + SNP 2 mg/kg and LPS + SNP 3 mg/kg versus LPS group (*p* < 0.05 and *p* < 0.001, respectively). Furthermore, total thiol concentration in LPS + SNP 3 mg/kg had a higher level than LPS + SNP 1 mg/kg and LPS + SNP 2 mg/kg groups (both *p* < 0.001) (Figure 6b).

SOD activity was also significantly different among the groups in the hippocampi (*F*_(4,30)_ = 51.44, *p* < 0.001). A significant difference was also seen among the groups in CAT activity in the hippocampi (*F*_(4,30)_ = 38.22, *p* < 0.001). The findings elucidated that LPS could reduce the activity of these enzymes in hippocampi tissues in LPS group in comparison to control group (both *p* < 0.001). Administration of SNP at a dose of 2 mg/kg increased SOD activity in the hippocampi of LPS + SNP 3 mg/kg group in compared to LPS group (*p* < 0.001), Use of 2 and 3 mg/kg of SNP before LPS boosted the CAT activity in LPS + SNP 2 mg/kg and LPS + SNP 3 mg/kg groups in comparison to LPS group (*p* < 0.01–*p* < 0.001). The activity of CAT and SOD in LPS + SNP 3 mg/kg groups was higher that LPS + SNP 1 mg/kg and LPS + SNP 2 mg/kg groups (*p* < 0.001) (Figure 6c,d).

#### 3.2.3 Results of MDA and thiol concentration and SOD and CAT activity in the cortex

**FIGURE 7:**
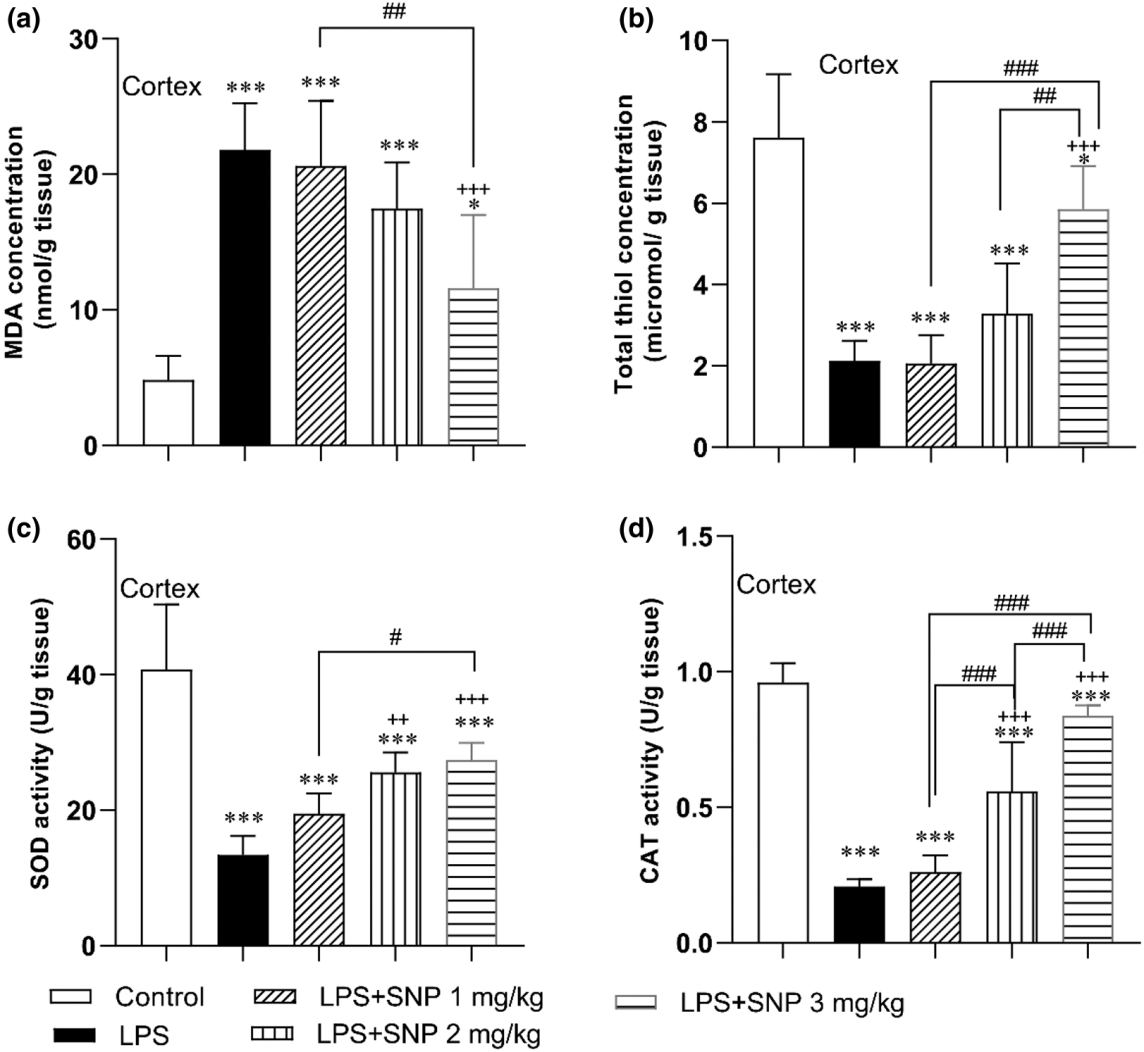
Comparison of the MDA (a) and thiol (b) concentration and SOD (c) and CAT (d) activity in cortex tissues between experimental groups. Data are presented as mean ± SD (*n* = 7 in each group). *p < 0.05 and ***p < 0.001 compared to control group. ^++^ *p* < 0.01, and ^+++^ *p* < 0.001 compared to LPS group. ^#^ *p* < 0.05, ^##^ *p* < 0.01, and ^###^ *p* < 0.001 compared to the other treatment groups.

MDA concentration in cortex had a considerable difference among the groups (*F*_(4,30)_ = 22.07, *p* < 0.001). The results also exhibited that LPS heightened the content of this product in cortex tissue of rats versus control group (*p* < 0.001). MDA concentration in the cortex tissue of LPS + SNP 3 mg/kg group was lower as compared with LPS group (*p* < 0.001) (Figure 7a). In addition, the MDA content in cortex tissue of LPS + SNP 3 mg/kg group was lower than LPS + SNP 1 mg/kg group (*p* < 0.01) (Figure 6a). The thiol level in the cortex had a considerable difference among groups (*F*_(4,30)_ = 36.74, *p* < 0.001). As illustrated in Figure 7b, LPS administration reduced the level of total thiol in the cortex of LPS group versus control group (*p* < 0.001). This antioxidant parameter was significantly increased in the cortex tissue of rats in LPS + SNP 3 mg/kg versus LPS group (*p* < 0.001). Furthermore, total thiol concentration in LPS + SNP 3 mg/kg had a higher level than LPS + SNP 1 mg/kg and LPS + SNP 2 mg/kg groups (*p* < 0.001 and *p* < 0.01, respectively) (Figure 7b).

SOD activity was also significantly different among the groups in the cortex (*F*_(4,30)_ = 29.30, *p* < 0.001). A significant difference was also seen among the groups in CAT activity in the cortex (*F*_(4,30)_ = 88.42, *p* < 0.001). The findings elucidated that LPS could reduce the activity of these enzymes in the cortex tissues in LPS group in comparison to control group (*p* < 0.001). Use of 2 and 3 mg/kg of SNP before LPS boosted the SOD and CAT activities in LPS + SNP 2 mg/kg and LPS+ SNP 3 mg/kg groups in comparison to LPS group (*p* < 0.01–*p* < 0.001). The activity of SOD in LPS + SNP 3 mg/kg group was higher that LPS + SNP 1 mg/kg group (*p* < 0.05). The activity of CAT in LPS + SNP 2 mg/kg and LPS + SNP 3 mg/kg groups was higher that LPS + SNP 1 mg/kg group (*p* < 0.001). The activity of CAT in LPS + SNP 3 mg/kg groups was higher that LPS + SNP 2 mg/kg group (*p* < 0.001) (Figure 7c,d).

## 4 DISCUSSION

The finding elucidated that LPS disturbed learning and memory in rats. In MWM task, long time and distance for searching the hidden platform by rats of LPS group was seen. In probe day, LPS also caused that the rats could not act successfully in remembering the location of the platform. In PA task, memory retention of LPS group to enter the dark room after receiving the electrical shock was weaker than control group. The latency time to enter the dark room in LPS group was shorter, whereas, time spent in the dark room was longer than control group. Findings are concordant with the results of previous studies that LPS injection impaired cognitive behaviors and synaptic plasticity in rats (Anaeigoudari et al., 2015; Lee et al., 2008; Pourganji et al., 2014).

Inflammation and oxidative stress are two important factors which can disturb brain function, cognition, learning and memory (Anaeigoudari et al. 2016; Hosseini et al., 2012). LPS provokes inflammatory reactions and ultimately impairs learning and memory (Bak et al., 2013; Anaeigoudari et al. 2016). It has been recognized that the administration of LPS into brain ventricles or its systemic injection impaired learning and memory in rats via enhancing the level of TNF‐α, IL‐1β, and IL‐6 (Arab et al., 2022; Hassanzadeh‐Taheri et al., 2021). Ullah et al. (2022) also reported that neuro‐inflammation resulted from LPS injection disturbed cognitive functions in mice. In addition, Amooheydari et al. (2022) suggested intraperitoneal administration of LPS stimulated lipid peroxidation and decreased the level of total thiol in hippocampus and cerebral cortex of mice and consequently unsettled spatial memory. In this study, besides increasing the brain levels of IL‐6, LPS incremented the MDA concentration, reduced the content of total thiol and SOD and CAT activity in hippocampi and cortex tissues of rats. These effects of LPS were accompanied with the impairment in spatial learning and memory retention.

SNP is a NO donor which is mainly employed in hypertension treatment (Titulaer et al., 2022). SNP has been also recommended to alleviate schizophrenia symptoms via affecting the N‐methyl‐D‐aspartate receptor signaling (Shim et al., 2016). Huang et al. (1995) reported that injection of moderate doses of SNP into dentate gyrus enhanced retention performance in a dose‐response manner in rats, whereas high doses of it disturbed memory retention in PA task. It has been shown that SNP also induced a long lasting enhancement in synaptic efficacy similar to LTP in rat's hippocampus (Böhme et al., 1991). SNP also could reverse aging‐caused memory impairment in rats through elevating the level of NO in hippocampus (Paul et al., 2005). Additionally, use of nitric oxide synthase inhibitors could ameliorate cognitive disturbance resulting from LPS in rat (Anaeigoudari et al., 2015; Anaeigoudari et al., 2016; Anaeigoudari, Soukhtanloo, Reisi, Beheshti, & Hosseini, 2016). In current research injection of SNP also ameliorated LPS‐caused memory dysfunction in rats. In MWM test, the rats of LPS‐SNP 2 mg/kg and LPS‐SNP 3 mg/kg groups not only spent shorter time and traveled less distance to reach the platform but also looked for the location of platform better in probe day than those of LPS group. In PA test also all three doses SNP prolonged the latency time to enter the dark camber and time spent in light room and shortened time spent in dark room in rats of LPS–SNP groups versus LPS group.

Anti‐inflammatory properties of SNP have been confirmed. For example, cardio‐protective effect of SNP against inflammatory reaction has been confirmed (Freyholdt et al., 2003). In addition, it has been reported that SNP could protect kidney (Sánchez‐Pérez‐Verdía et al., 2001) and lung (Anaya‐Prado et al., 2004) against injuries resulted from ischemia‐reperfusion via suppressing the expression of inflammatory mediators and leukocyte–endothelium adhesion. SNP has also been revealed to be able to decrease LPS‐induced COX‐2 expression via inhibiting the nuclear factor kappa B (NF‐κB) binding to DNA in monocytes (D'Acquisto et al. 2001). In agreement with these findings, in present study SNP decreased the serum level of IL‐6 in LPS–SNP groups in comparison to LPS group.

In addition, antioxidant effect of SNP also has been documented (Ortuño‐Sahagún, 2013). SNP has been demonstrated to be able to decrease oxidative stress by inhibiting lipid peroxidation (Khan et al., 2006). In this study, SNP especially at dose 3 mg/kg improved LPS‐induced oxidative stress through decreasing MDA concentration and enhancing total thiol content and SOD and CAT activities in rat's hippocampi and cortex tissue. Therefore, it can be concluded that improving effects of SNP on memory deficits caused by LPS probably has been mediated via reducing inflammation and oxidative stress.

Furthermore, it should be considered that NO‐cGMP‐PKG pathway is an important signaling pathway involving in induction of learning and memory processes (Chien et al., 2008). It should also be kept in mind that LPS decreases the cellular level of cGMP via inhibiting guanylyl cyclase and activating phosphodiesterase (El‐Awady et al., 2008), whereas, SNP increases the cGMP generation (Hallak et al., 2013). Therefore, it is possible that the activation of this path way by SNP is involved in results of current study. However, all these mechanisms require further research to be clarified.

In summary, LPS impaired spatial learning and memory retention. SNP reversed the harmful effects of LPS on learning and memory. Considering the biochemical results, it is possible that the positive effect of SNP against memory dysfunction induced by LPS is mediated via modulating inflammatory responses and brain oxidative stress.

## FUNDING INFORMATION

The authors would like to appreciate Vice Presidency of Research of Mashhad University of Medical Sciences for their support (991876).

## CONFLICT OF INTEREST STATEMENT

No conflict of interest has been advertised.

## ETHICS STATEMENT

All tests were done under guidlines of Ethical Commitee of Mashhsd University of Medical Sciences (IR.MUMS.MEDICAL.REC.1400.094).

## Data Availability

The authors will present the data whenever needed.
